# Anthocyanin accumulation enhanced in *Lc*-transgenic cotton under light and increased resistance to bollworm

**DOI:** 10.1007/s11816-015-0382-3

**Published:** 2015-12-10

**Authors:** Xiaoping Fan, Bohong Fan, Yuxiang Wang, Weicai Yang

**Affiliations:** Institute of Cotton Research, Academy of ShanXi Agricultural Science, Yuncheng City, Shanxi Province 044000 China; Key Laboratory of Molecular and Developmental Biology and National Centre for Plant Gene Research (Beijing), Institute of Genetics and Developmental Biology, Chinese Academy of Sciences, Datun Road, Beijing, 100101 China

**Keywords:** *Lc* gene, *Gossypium hirsutum*, Anthocyanin, Cotton fiber

## Abstract

Breeding of naturally colored cotton fiber has been hampered by the limited germplasm, an alternative way is to use transgenic approach to create more germplasm for breeding. Here, we report our effort to engineer anthocyanin production in cotton. The maize *Lc* gene, under the control of the constitutive 35S promoter, was introduced into cotton through genetic transformation. Our data showed that the expression of the *Lc* gene alone is sufficient to trigger the accumulation of anthocyanin in a variety of cell types including fiber cells in cotton. However, the accumulation of colored anthocyanin in cotton fibers requires the participation of light signaling. These data indicate that it is feasible to engineer colored fibers through transgenic approach in cotton. Furthermore, we showed that the *Lc*-transgenic cotton plants are resistant to cotton bollworm. These transgenic plants are, therefore, potentially useful for cotton breeding against cotton bollworm.

## Introduction

Naturally colored cotton clothing and textile products become more and more popular in people’s life. They not only have the same advantages as the common white fiber, but also require no chemical dyeing and bleaching. Therefore, they are environmental friendly as well. Early in 1990s, Bird Brothers successfully bred four colored fibers: green, brown, red (a reddish brown) and mocha (similar to tan). However, so far breeding of naturally colored cotton fiber is limited by the lack of proper germplasm, which is largely limited to brown and green fiber traits. Furthermore, genetic studies on genes controlling pigment production in cotton have been hampered by its complex genome. Therefore, it is necessary to find more cotton germplasm that introduces novel color traits into cotton through transgenic approaches, for example, by modifying anthocyanin biosynthetic pathway.

Biosynthetic pathway leading to anthocyanin pigmentation has been studied extensively in *Zea mays* and genes controlling each step have been characterized (Sharma et al. 2012). Anthocyanin biosynthesis is controlled by two groups of genes. One group is the structural genes which encode the enzymes, catalyzing the biochemical reactions of the formation of anthocyanins. The second group includes genes which encode transcription regulators that control the expression of the structural genes, and determine the spatial and temporal pattern of anthocyanin accumulation. In general, the transcription regulators belong to either the MYB-type C1 family or the basic helix-loop-helix, MYC-type R family (Dooner et al. 1991; Boudet 2007; Kong et al. 2012). Both *C1* and *R* genes have been studied extensively in many plants, such as maize (Kong et al. 2012) and petunia (Quattrocchio et al. 1993; Schwinn et al. 2014). The *C1* gene of maize plays a regulatory role in the production of anthocyanin pigments in the aleurone layer of the endosperm (Cone et al. 1986; Stinard et al. 2009) and the *R* genes control the temporal and spatial distribution of anthocyanin pigments (Ludwig et al. 1989).

The *Lc* (leaf color) gene is a bHLH (basic/helix-loop-helix) anthocyanin regulator of maize, belongs to the *R* gene family, determines the timing (Ludwig and Wessler 1990), distribution, and amount of anthocyanin pigmentation in maize and is the target for plant pigment engineering (Schwinn et al. 2014). In this family, it also consists of other regulatory genes, such as *Sn* and the B locus genes (Chandler et al. 1989; Dooner et al. 1991; Consonni et al. 1993; Petroni and Tonelli 2011). The *Lc* gene as a transcription factor can regulate the anthocyanin biosynthesis pathway and use anthocyanin-regulating transcription factors to alter anthocyanin production and pigmentation patterns in several plant systems (Boase et al. 1998; Bradley et al. 1998), such as in tomato (Goldsbrough et al. 1996; Bovy et al. 2002), tobacco (Lloyd et al. 1992; Huang et al. 2012), apple (Flachowsky et al. 2010) and petunia (Bradley et al. 1998; Albert et al. 2009). In transgenic petunia plants, the maize *Lc* gene, under the control of the constitutive CaMV 35S promoter, enhanced pigmentation in both vegetative and floral tissues. Additionally, in *Lc*-transgenic alfalfa foliage, red–purple colored anthocyanin accumulated under high light conditions (Ray et al. 2003). These reports illustrated that anthocyanin production in vegetative and reproductive tissues was enhanced and induced by *Lc* transcription factors under high light conditions (Procissi et al. 1997; Albert et al. 2009). However, in several different plant species, anthocyanin biosynthesis was stimulated by *Lc* and C1 together (Han et al. 2009), or in some species *Lc* has no visible effect (Boase et al. 1998; Bradley et al. 1999). In cotton, few studies on *Lc* transformation and expression have been reported so far. Therefore our aims for this study were to determine whether the *Lc* gene alone can active anthocyanin biosynthesis in cotton and characterize the anthocyanin pigmentation response in *Lc*-transformed cotton plants.

For another, anthocyanin in leaves has been correlated with resistance to biotic and abiotic agents, including fungi, herbivores and cold (Chalker-Scott 1999; Gould et al. 2000; Huang et al. 2012). The anthocyanin biosynthetic pathway is interlinked with the biosynthesis of flavonoid compounds, which are important to fertility and defense against insects and pathogens (Dooner et al. 1991; Flachowsky et al. 2010). Red leaves are less frequently attacked than green leaves; in *Lc*-transgenic rice, red leaves exhibited increased resistance to blast infection (Gandikota et al. 2001). Therefore, expression of *Lc* provides us a tool to engineer cotton color and insect resistance. Here, we report our approach to employ *Lc* gene to engineer cotton color and insect resistance.

## Materials and methods

### Plant materials

Cotton (*Gossypium hirsutum*) Coker 312 seeds were surface sterilized by soaking for 1 min in 70 % ethanol followed by ~2 h in 10 % H_2_O_2_. They were then washed with sterilized distilled water 3 times. Seeds were allowed to germinate in sterilized water for 16–20 h at 28 °C. The germinated seeds were transferred to half strength MS medium (Murashige and Skoog 1962) containing 15 g/L glucose and 3 g/L Phytagel. The pH was adjusted to 5.8 with 1 M KOH prior to autoclaving. The transformation procedure was carried out as described by Li et al. (2005). Briefly, hypocotyl explants of 5- to 7-day-old seedlings were cut into 0.8–1-cm-long segments and immersed in a bacterial culture suspension with gentle agitation. Then, the co-culturing, selection, tissue culturing, differentiation, germination and rooting processes were performed as described previously (Li et al. 2005). All tissue culture processes before transplanting to pots were performed in a culture room that was maintained at 28 °C with a 16/8 h light/dark photoperiod and light supplied by warm-white fluorescent lamps at an irradiance of 26 µmol/m^2^/s.

### Bacterial culture and cotton genetic transformation

*Agrobacteriumtumefaciens* strain LBA4404, which contains a modified plasmid pBI121 harboring a CaMV 35S::Lc::Nos transgene (provided by Dr. Lim Saw Hoon, Malaysia University of Science and Technology, Malaysia), was cultured overnight in LB liquid medium with continuous shaking at 250 rpm, at 28 °C. Bacterial cultures at OD_600_ 0.6–0.8 were collected by centrifugation and resuspended in MS liquid medium to a final OD_600_ of 0.2–0.4. Acetosyringone was added to the culture medium to a final concentration of 100 μM at ~2 h before harvesting. Cotton transformation and tissue culturing were performed as described previously (Li et al. 2005).

### Screening of transgenic plants using polymerase chain reaction (PCR)

The analysis of the transgenic lines of the T0 and T1 generations was carried out by amplifying a fragment of the *Lc* gene using PCR. Genomic DNA was extracted from young leaves of greenhouse-grown transgenic and nontransgenic plants using the CTAB method (Li et al. 2002). PCR screening of putative transformants was performed using primers specific to the *Lc* gene. The plasmid was used as a positive amplification control. The sequences of the forward and reverse primers are 5′ATGGCGCTTTCAGCTTCCCGAGT3′ and 5′TCACCGCTTCCCTATAGCTTTGC3′, respectively. The PCR master mix was made according to the PCR kit’s protocol. Amplification was accomplished using a Thermal Cycler (Biometro, Germany). The reaction was as follows: incubation at 94 °C for 5 min, and then a step cycle program set to denature at 94 °C for 45 s, to anneal at 58 °C for 45 s, and then extended at 72 °C for 2 min, for a total of 35 cycles, followed by a final extension at 72 °C for 10 min.

### Southern blot analysis

For the Southern blot analysis, 50 μg genomic DNA was isolated from PCR-positive plants and digested with 20 units of *BamH*1 at 37 °C overnight in a total volume of 200 μl. Digested DNA was gel fractionated and transferred onto Hybond N+ nylon membrane (Amersham) according to the manufacturer’s instructions. The *Lc* probe was labeled with Digoxigenin-11-dUTP using PCR amplification according to the manufacturer’s instructions (Roche). Hybridization was performed overnight at 42 °C using DIG-Easy-hybridization solution containing a DIG-labeled *Lc* probe. The hybridized membrane was washed 3 times with a 0.1× SSC plus 0.1 % SDS solution at 68 °C and detection was carried out using an anti-DIG-AP conjugate according to the manufacturer’s instructions.

### RNA isolation and reverse-transcription (RT)-PCR analysis

The fresh young leaves, petiole and floral tissues, including petal, stigma, anther and sepal, in addition ovule, fiber at the proper development stage, were collected and frozen in liquid nitrogen. Total RNA from these materials was extracted using the hot phenol extraction method (Li et al. 2005). Then, 1 µg of each total RNA sample was reverse transcribed using the Qiagen one-step RT-PCR protocol. Briefly, the RNA template was added to the master mix and reverse transcribed at 50 °C for 30 min. Then, the reverse transcriptase was inactivated, and the cDNA template was denatured at 95 °C for 15 min. PCR amplification was performed using *Lc* gene-specific primers, and a program of 30 cycles of 94 °C for 45 s, 56 °C for 45 s, and 72 °C for 2 min, followed by a final extension at 72 °C for 10 min. The cotton *actin* gene was used as an internal standard to normalize the cDNA concentration. The same amplification was performed using the *actin* primers: Actin-F, 5′TTTGCTGGTGATGATGCTCC3′; and Actin-R, 5′CTCCAATCCAGACACTGTACT3′.

### Ovule culture

Cotton bolls from 2-day post-anthesis T1 CaMV 35S::Lc::Nos transgenic plants of two lines, L73 and L143, and wild type (Wt) were surface sterilized in 70 % ethanol for 5–10 min, and then immature ovules were gently transferred onto refined liquid medium as described by Beasley and Ting (1974). Immature ovules from half of one boll were cultured under a 16/8 h light/dark photoperiod, while the other half were cultured in complete darkness in a culture room. Same stage ovules from non-transgenic bolls were cultured under the same conditions. The comparative experiment was repeated six times.

### Anthocyanin extraction and measurement

The anthocyanin extraction was performed according to Choung et al. (2001). Air-dried old or fresh leaves were ground to a powder. Then, 5 g of powder was extracted with 100 ml of a 1 % HCl and 40 % methanol mixture in the darkness at 4 °C overnight. Subsequently, the extracts were centrifuged and filtrated through a 0.45 μm filter. The anthocyanin pigment content was determined by a pH differential method commonly used in food technology (Gong et al. 1997). Briefly, 10 ml of extract was adjusted to pH 1.0 and another 10 ml to pH 4.5 with 0.025 M potassium chloride buffer and 0.4 M sodium acetate buffer, respectively. Then, the extracts were analyzed using a UV–VIS spectrophotometer and quantified (Gong et al. 1997). The anthocyanin content, expressed as cyanidine-3-glucoside, was determined according to Lambert–Beer’s Law: *A* = *εC*Len (*A*, absorbance; *C* concentration; Len, path length in cm; and *ε*, molar extinction coefficient) using the molar extinction coefficient of 26,900 Len/mol and molar mass of 449 g/mol.

The color density, polymeric color and the percent of tannin contribution were measured using the bisulfite solution method (Somers and Evans 1974). The metabisulfite is able to bleach the anthocyanin, but not polymeric tannin pigments, and spectrophotometric assay results were calculated according to Cortell et al. (2007). The percent contribution of tannin is the ratio of polymeric color to color density.

### Insect bioassay

The 4th leaf from the apical shoot of each line (L73, L74, L143, L144 and Wt) picked for the bioassay was washed thoroughly with sterile distilled water prior to feeding. Both transgenic red leaves and non-transgenic leaves at the same distance from shoot apex were placed in one petri dish and infested with three-third-instar cotton bollworm larvae (*Helicoverpa armigera*) that had been starved for 2 h. After 3 days, the feeding preference was recorded, and 13 replicate samples from each line were used.

In addition, the third-instar larvae were placed into one petri dish with either a piece of transgenic or non-transgenic leaf, observed every day and weighed after 5 days of feeding. The insect weights on different plant lines were determined using the between-column variance method. Larval mass (to an accuracy of 0.1 mg) was measured. To compare herbivore resistance-associated parameters between Wt and *Lc*-transgenic lines a one-way analysis of variance (ANOVA) was performed, and the experiment was replicated three times.

## Results

### Generation of transgenic cotton plants

**Fig. 1:**
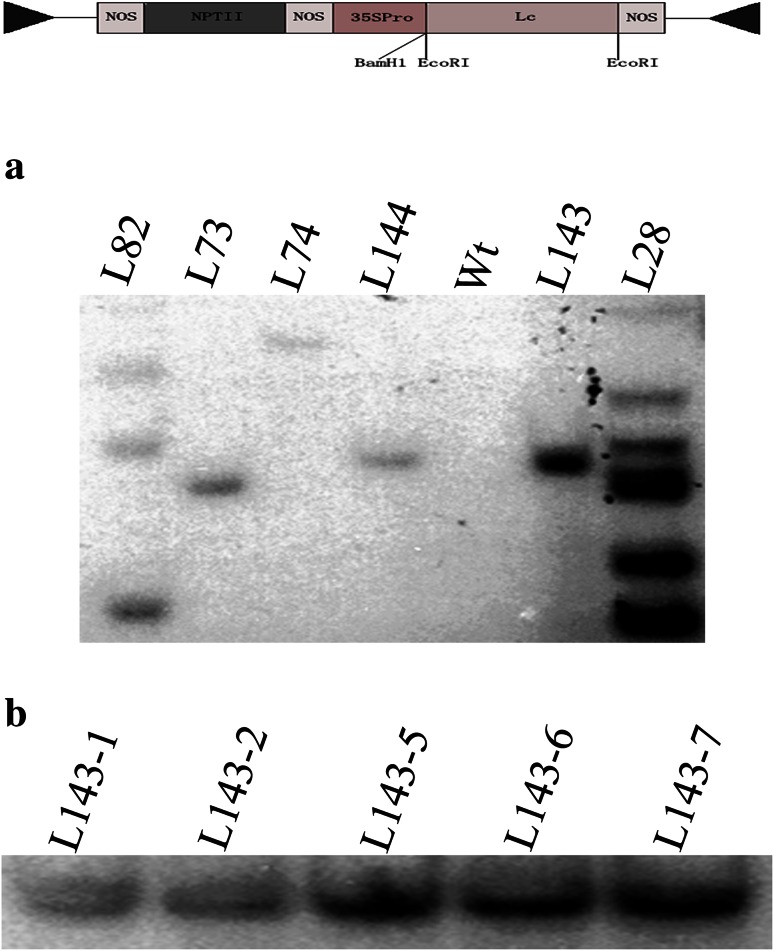
Southern blot analysis of the *Lc*-transgenic cotton plants. Genomic DNA from T0 transgenic (**a**) and T1 transgenic plants (**b**) was digested by *BamH*I and fractionated using gel electrophoresis. The blot was probed with a DIG-labeled *Lc* probe. **a** Lanes *1*–*4* and *6*–*7* represent six independent transgenic lines as indicated. Lane *5* is a nontransgenic control (Wt). **b** Lanes *1*–*5* represent five T1 siblings from L143 showing *Lc* hybridization bands

After transformation and regeneration from tissue culture, through somatic embryogenesis, six independent transgenic lines were regenerated on *kanamycin* selective medium. To confirm the presence of the *Lc* transgene in these T0 transgenic lines, genomic DNAs were digested by *BamH*I and fractionated by gel electrophoresis. The blot was probed with a DIG-labeled *Lc* probe. Six PCR positive plantlets were confirmed by Southern hybridization. Southern blot hybridization analysis determined transgene copy numbers from these lines. Lines of L82 and L28 had multiple copies insertion and the other four lines (L73, L74, L143 and L144) had single insertion, respectively. The non-transgenic control (NC) lacks the *Lc* hybridization signal (Fig. 1a). Southern blot analysis was performed on the T1 generation to reveal the *Lc* gene information of integration and separation in T1 generation. In line L143 T1 generation, 5 (L143-1, 2, 5, 6 and 7) of 15 were positive (Fig. 1b).

### *Lc* gene expressed at the molecular level

**Fig. 2:**
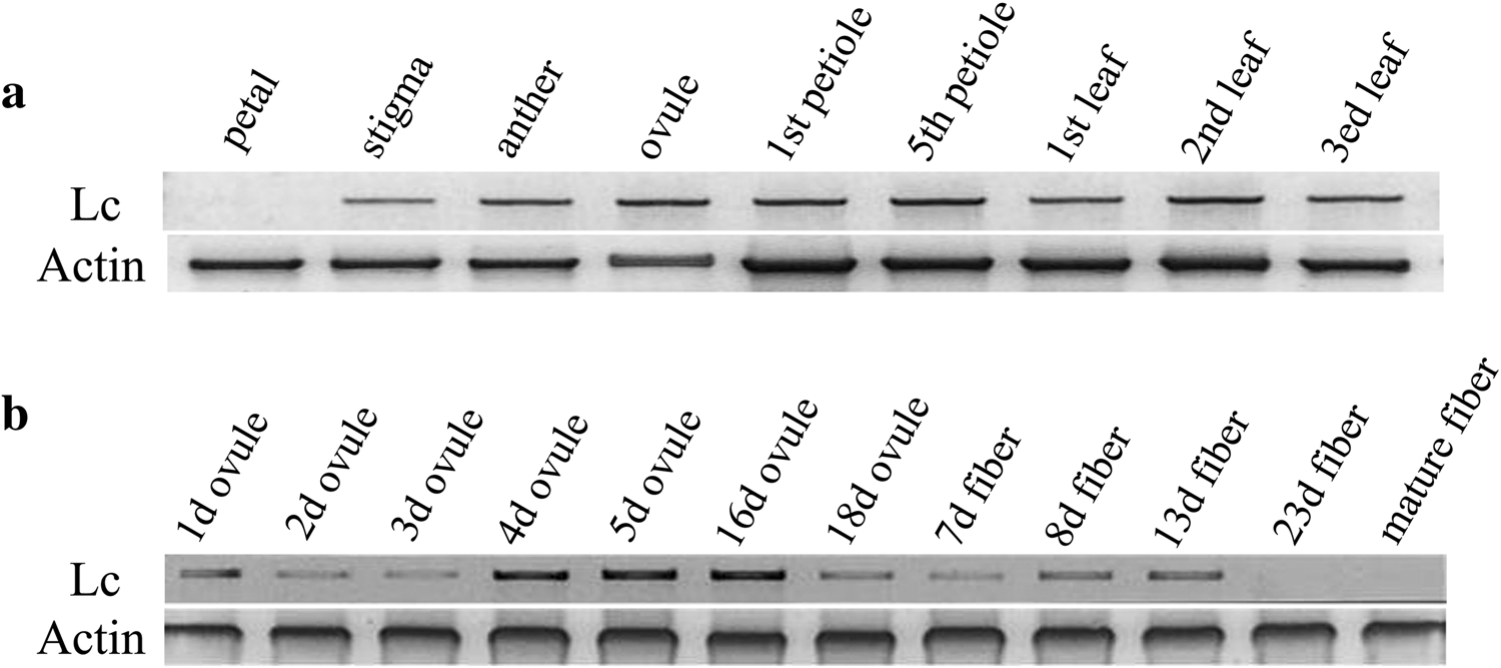
RT-PCR analysis of *Lc* expression patterns in transgenic cotton plants. **a** *Upper panel* *Lc* expression level in the indicated vegetative and reproductive tissues; *lower panel* *Actin* was used as internal control indicating no DNA contamination in the samples. **b** *Lc* expression in ovules and fibers at the indicated stages. *Actin* was used as an internal control

To investigate whether the *Lc* transgene is expressed in the transgenic plants, RT-PCR was performed from different tissues and organs. Our results showed that *Lc* was expressed in leaves and floral tissues of the transgenic plants, and no *Lc* transcripts were detected in non-transgenic plants (data not shown). The expression levels of the *Lc* gene varied slightly among tissues and organs (Fig. 2a). Leaves and petioles had relatively higher *Lc* expression levels than the floral tissues. Additionally, the *Lc* expression levels were different among flower parts. The highest expression level was in anther, while it was moderate in stigma, but no signal was detected in petals.

The *Lc* gene was expressed during all the ovule and fiber developmental stages in the transgenic plants. There were high expression levels in the 4–16 DPA (days post-anthesis) ovules, and moderate levels were detected in 1–3 DPA ovules and 18 DPA ovules. To determine whether the *Lc* transgene was expressed only in cotton fiber cells, we isolated RNA from fibers of 7, 8, 13 and 23 DPA ovules and mature fibers, respectively. The test results showed that in fiber, the expression level rose along with fiber development from 7 to 13 days. The expression level decreased to a weak but detectable signal in 23 DPA fibers and was weaker in mature fiber cells (Fig. 2b).

### Morphology of *Lc*-transgenic cotton

**Fig. 3:**
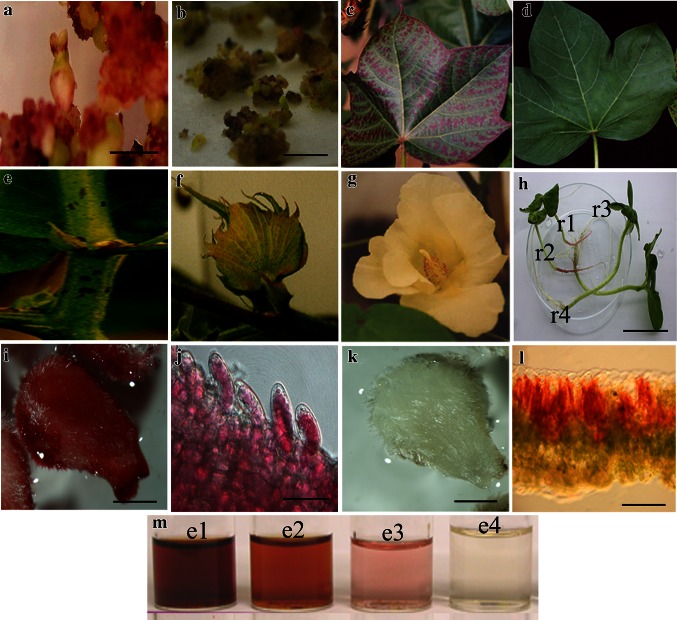
Phenotypic characterization of *Lc*-transgenic cotton. **a** Red transgenic calluses and somatic embryos (*bar* 2.5 mm). **b** Non-transgenic calluses (*bar* 2.5 mm). **c** Transgenic *purple colored* leaf, indicating anthocyanin accumulation. **d** Non-transgenic leaf. **e** Anthocyanin accumulation on stem (*red patches*). **f** Transgenic flower bud with a red sepal. **g** Transgenic flower with a red anther. **h** T1 siblings showing the segregation of *Lc*-dependent anthocyanin accumulation in roots. r1 and r2 are positive seedlings with red roots; r3 and r4 are negative seedlings with white roots. **i** Transgenic immature ovules cultured under light for 1 week (*bar* 2 mm). Note red fiber cells. **j** Magnification of J showing a single red fiber cell (*bar* 10 μm). Note red oily liquid gathered in the central big vacuole. **k** Transgenic immature ovules cultured in completely dark for 1 week (*bar* 2 mm). **l** Transgenic leaf abaxial surface toward the sun; its freehand cross section with red palisade mesophyll cells. The epidermal cells are colorless and transparent (*bar* 1 mm). **m** Anthocyanin extract from transgenic dry leaves (e1), transgenic fresh leaves (e2), Wt leaves (e3), and empty control (e4)

During the regeneration process, most of the transformants accumulated high levels of anthocyanin pigment in their vegetative and reproductive tissues. In the tissue culture process, we observed that *kanamycin*-resistant calluses and somatic embryos appeared an unusual red color (Fig. 3a) that was never observed in control tissues (Fig. 3b). Generally, anthocyanin pigmentation accumulated in radicles, hypocotyls and cotyledons of transgenic somatic embryos. The purple color present in cotyledons disappeared several days later, and the young root turned red gradually in a culture bottle. The red root phenotype never presented in non-transgenic plants. Juvenile leaves usually displayed red, when the transgenic plantlets were transplanted into soil. Some appeared purple in pigmentation when the leaves unfolded (Fig. 3c), and Wt leaves were green under the same growth conditions (Fig. 3d). With further growth of the regenerated plants, some red patches appeared on the stem’s surface (Fig. 3e). The sepal around the flower bud turned red (Fig. 3f) when the flower opened, and the anthers and the stigma also appeared red (Fig. 3g). We observed that the anthers are larger and contain less pollen compared with the Wt. However, no anthocyanin pigmentation was observed in petals at the day of anthesis. We found that the old leaves of transgenic plants turned deep red, and their air-dried leaves were more fragile when squeezed. Non-transgenic senescent leaves remained yellowish and were more flexible after air drying. The L143 and L73 lines were fertile, and their T1 and T2 generations had the same red color characteristics. The T1 generation segregated 3:1 (positive: negative). The positive seedlings had red roots (Fig. 3h-r1, r2) and the negative ones were white when germinated in a bottle under the same culture conditions (Fig. 3h-r3, r4).

### Light-induced anthocyanin accumulation in transgenic fibers and leaves

The initiating fibers of transgenic ovules became red (Fig. 3i) after being cultured in vitro under light for 7 days, and a red oil-like liquid accumulated in the large central vacuole of the single fiber cell observed under the microscope (Fig. 3j). Fiber cells of non-transgenic ovules at the same developmental stage showed little color change on the fiber surface under the same light (data not show). When cultured in the dark, the other areas, the fiber no matter from transgenic or non-transgenic bolls, remained completely white (Fig. 3k). Light is also an important factor that affects anthocyanin production in leaves. Freehand cross section of leaves from transgenic plants grown with exposure to normal sunlight for 3 days exhibited anthocyanin accumulation in their palisade mesophyll cells (Fig. 3l). When the upper surface of the leaf was not exposed to sunlight, for example with positioning facing down (due to abnormal growth), the spongy mesophyll cells turned red but the palisade mesophyll cells remained green (data not shown). The leaf epidermis was always maintained colorless and transparent whichever side faced toward the sun (Fig. 3l).

### Transgenic plants produce more anthocyanin than non-transgenic plants

We further quantified the amount of anthocyanin in transgenic plants. The leaf extract of transgenic dry or fresh leaves showed more intense red (Fig. 3m-e1, e2) compared with the Wt leaves (Fig. 3m-e3). Figure 3m-e4 shows the empty control.

To quantify the amount of anthocyanin, we employed the pH differential method commonly used in food technology. Measurement results showed that the anthocyanin, exemplified by cyanidin-3-glucoside, increased from 15.41 mg/100 g (Wt) to 53.60 mg/100 g (*Lc*) in the dry leaf extract and from 9.74 mg/100 g (Wt) to 12.80 mg/100 g (*Lc*) in the fresh leaf extract.

**Fig. 4:**
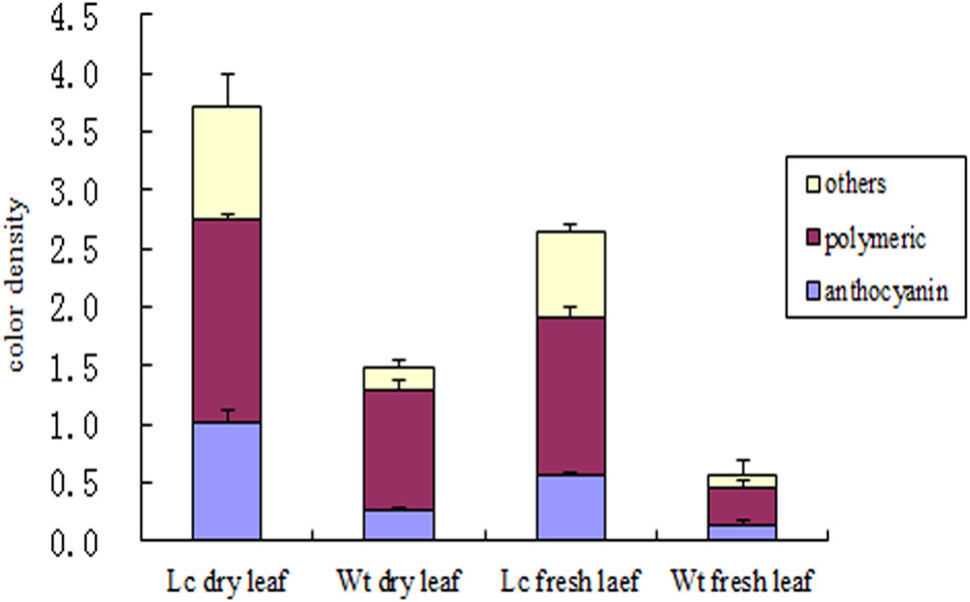
Anthocyanin content revealed by UV spectrometric analysis. Relative fractions of monomeric anthocyanin, polymeric anthocyanin (condensed tannins) and other compounds of the total anthocyanin calculated using the bisulfite leaching method. *Values* represent averages ± STEV, and every *bar* represents an average value from three repeated measurements

Using the bisulfite solution method, anthocyanin degradation indices data showed that the three indices, color density (total anthocyanin), and monomeric and polymeric anthocyanin (condensed tannin) (Cortell et al. 2007) increased when compared with Wt extract. Color density in *Lc*-transgenic leaf rose from 1.62 to 4.03 in the dry leaf extracts; in fresh leaf extract, it increased from 0.71 to 2.76. However, the ratio varied between monomeric anthocyanin and polymeric anthocyanin. In *Lc*-transgenic dry leaf, monomeric anthocyanin accounted for 27.2 % of the total anthocyanin relative to 17.3 % in non-transgenic dry leaf. Polymeric anthocyanin ratio from 67.2 % in Wt varied to 62.4 % of the total anthocyanin. In fresh materials, monomeric anthocyanin percentage decreased from 22.5 to 20.7 %, while the polymeric anthocyanin percentage increased from 50.7 to 51.4 % in transgenic leaf extract (Fig. 4). This might be due to the formation of polymers and anthocyanin degradation that could be occurring in *Lc* leaves during different stages of growth, leading to changes in the anthocyanin ratio.

### Transgenic plants are resistant to cotton bollworm

**Fig. 5:**
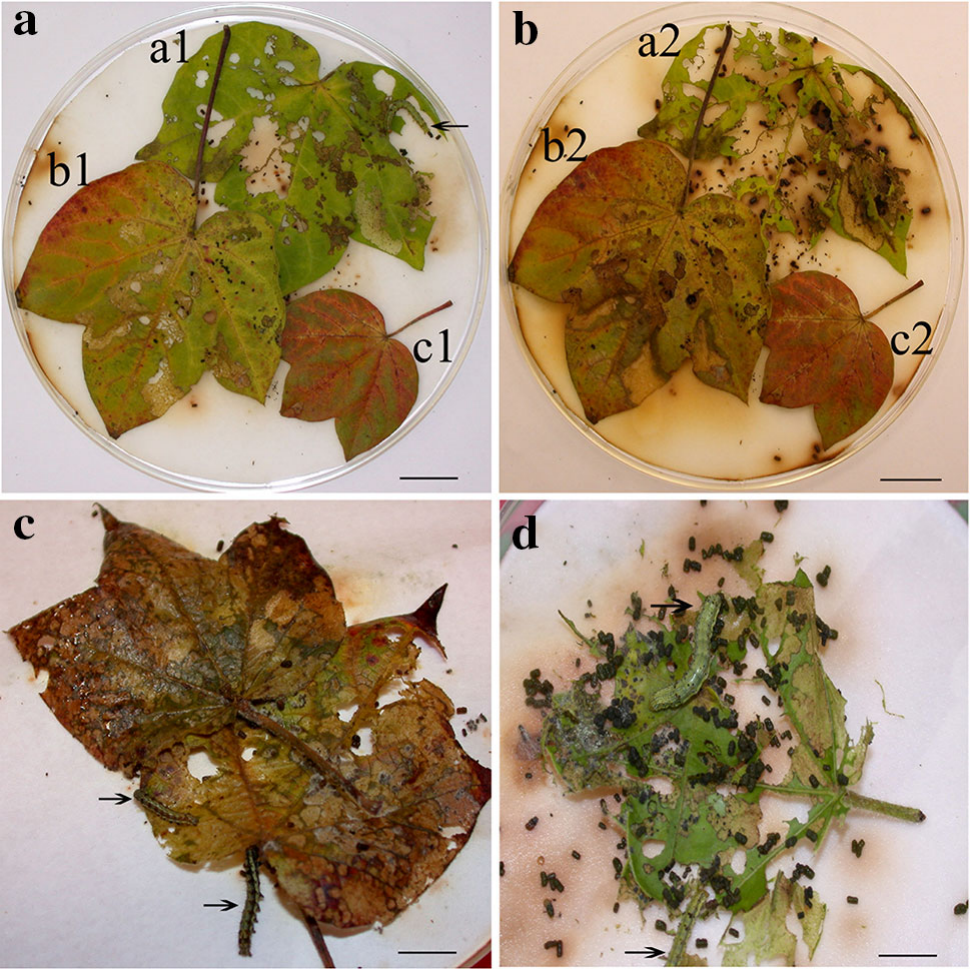
Bioassay for cotton bollworm feeding on *Lc* and Wt leaves. **a** Leaf images after 2 days of feeding. *b1* and *c1* are *Lc*-transgenic leaves, *a1* is a Wt leaf. The *arrow points* to worm (*bar*  2.2 cm). **b** Leaf images after 4 days of feeding. *b2* and *c2* are *Lc*-transgenic leaves, *a2* is a Wt leaf (*bar* 2.2 cm). **c** Image showing larvae after feeding 5 days on *Lc*-transgenic red leaves. The *arrows point* to worms (*bar* 2 cm). **d** Image showing larvae after feeding 5 days on Wt leaves. The *arrows point* to worms (*bar* 1.8 cm)

To test whether the CaMV 35S::Lc-transgenic plants were also resistant to cotton bollworm, an insect feeding experiment was performed. Our data showed that the insects fed preferably on non-transgenic leaves when both transgenic and non-transgenic leaves were present in one dish. The non-transgenic leaf was consumed more after 2 days of feeding (Fig. 5a-a1), while the transgenic leaves remained more (Fig. 5a-b1, c1). This trend became more obvious after 4 days of feeding (Fig. 5b-a2, b2, c2). Interestingly, the green part of the leaf was preferentially eaten by larvae compared with red patches on the same leaf.

**Fig. 6:**
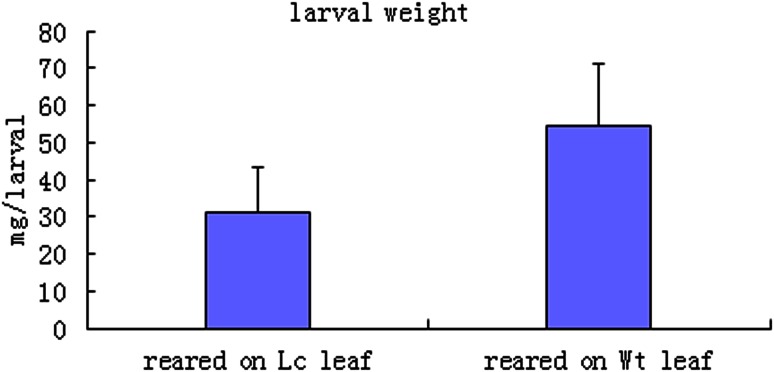
Statistical analysis of the larval weights after 3 days of rearing on *Lc*-transgenic and Wt leaves from Fig. 5b values are from 13 samples and represent averages ± STEV. Every *bar* represents the average weight repeated three times (*n* = 13)

A force-feeding assay is shown in Fig. 5; insect larvae that fed on transgenic leaves after 5 days (Fig. 5c) hardly ate and were immobile, and they were visibly smaller than those fed on non-transgenic leaves those were more active(Fig. 5d). To compare herbivore resistance-associated parameters between Wt and *Lc*-transgenic lines, the differences in larvae weight after feeding on Wt or *Lc*-transgenic leaves for 5 days, 13 groups data produced automatically a bar chart as Fig. 6 using Excel software, and was analyzed using a one-way ANOVA with SPSS statistical software. The analysis results are presented in Table 1; the *F* test value is 15.792, and *P* < 0.01. This illustrated that there was a significant difference between the larvae weights which fed on transgenic *Lc* leaves or non-transgenic leaves (Table 1). The larvae weight of those fed on non-transgenic leaves was significantly heavier than those fed on *Lc*-transgenic leaves. These data showed that expression of *Lc* confirms transgenic cotton resistance to cotton bollworms.

**Table 1:** Larval weights of cotton bollworms fed on *Lc*-transgenic leaves or Wt leaves were analyzed using a one-way ANOVA test by SPSS

	Sum of squares	df	Mean square	*F*	Sig
Between groups	3301.885	1	3301.885	15.792	0.001*
Within groups	5018.000	24	209.083		
Total	8319.885	25			

Statistical analysis of the larval weights (*n* = 13) after 3 days of rearing on *Lc* and Wt leaves. Average weight measurements were repeated three times (*n* = 13). *P* < 0.01* indicates that there was a significant difference between the two groups

## Discussion

### Expression of maize *Lc* promotes anthocyanin production in cotton under light

Our data showed that there were 6 lines of regenerated plants obtained from 16 lines of transgenic calluses. These plants were positive and confirmed by Southern blotting using *Lc* probe. Since there is only one *BamH*1 site in the T-DNA, the number of bands of hybridization is the T-DNA copy number of insertion into plant genome (Fig. 1a). From Fig. 1b, we find that the T-DNA was stably transmitted to the T1 generation and segregated in a 3:1 ratio as a single gene insertion in line of L143. In the positive transgenic plants, the maize *Lc* gene under the CaMV35S promoter enhanced pigmentation in both vegetative and most floral tissues except petals. During tissue culture process, it is evident that the color of the transgenic calluses varies from pink, red, dark red to dark purple due to the amount and the localized production of anthocyanin. Similar phenomenon has been observed in rice tissues (Gandikota et al. 2001). The purple colored calluses became more intense to dark purple during culture leading to no somatic embryos formed from them. Because too much anthocyanin or toxic flavonoids in this kind of calluses was detrimental to cells (Burbulis and Winkel-Shirley 1999; Gandikota et al. 2001). And those with slight pigment, red or pink colored calluses can continuously propagate to form somatic embryos. Purple or red colored calluses never have been seen in control untransformed cultures. Therefore, *Lc* gene could be used as a color-based selection marker for genetic transformation in cotton. In transgenic embryos, color intensity varies among embryos and no consistent pattern was observed. One type of embryos with slight pink color on hypocotyl, cotyledon and radicle can develop to maturity and germinate. Another type of embryos with dark purple around the whole body or most of the embryo were often arrested before the torpedo stage. Might anthocyanin accumulation have adverse effects on hypocotyl formation? Consistent observation has reported in rice that the maize *Lc* gene up-regulated the expression levels of various anthocyanin regulatory genes leading to sterility or abnormal seed development (Li et al. 2013). Anthocyanin production and pigmentation involve multiple steps and often display cell and tissue-specific patterns. In *Lc*-transgenic leaves, anthocyanin accumulates mainly in veins and palisade mesophyll cells rather than epidermis. There was stronger expression of signal in leaves and petioles. And the expression level increased slightly from the 1st leaf to the 3rd leaf. In reproductive tissues, although pigment and signal appeared in anther, sepal and stigma at the same stage in the same cotton flower, neither red color nor transcription signal was observed in the petals. This expression pattern was mainly determined by *Lc* gene in cotton rather than the CaMV 35S promoter which stimulated higher expression levels in all floral parts, including petals in cotton (Sunilkumar et al. 2002). Therefore, it is inferred that the *Lc-*activated pathway is under developmental control, and the transgene expression is regulated both spatially and temporally (Martin and Gerats 1993); like in *Lc*-transgenic tomato fruits, the reddish-purple pigmentation disappears as fruit ripening progresses (Bovy et al. 2002). Another reasoning based on studies in petunia may be that in the petals and anthers anthocyanin synthesis is controlled by two different ternary complexes (Petroni and Tonelli 2011). In the ovule or fiber cells, no regular pattern of transgene expression was found. Although stronger expression signal presented throughout the fiber developmental phase, the fibers still appeared white when the bolls opened. Is the color due to anthocyanin in cotton fibers going to last processing or will it fade when dried? Or anthocyanins are soluble pigment molecules and generally may not be stable in tissues after drying? To resolve this problem, we open the cotton bolls in every development stage, and found that the fiber was white all the way. Therefore, the test of ovule cultured in vitro under light was done. The light induction experiment showed that the white fiber changed to red color, and leaf turned redder, suggesting that light signaling is essential for the *Lc*-activated anthocyanin biosynthesis or the conversion of colorless intermediates into colored pigments in fibers and leaves, because light can influence the accumulation of anthocyanin primarily through the activation of the transcription factors that regulate the flavonoid biosynthetic pathway (Irani and Grotewold 2005). But it is not clear whether the light signaling activates additional genes essential for the conversion of colorless anthocyanin to colored anthocyanin, or enhanced *Lc* expression in fiber cells. Therefore breeding a red fiber germplasm needs to solve this problem of having to increase anthocyanin accumulation in the fiber without having to culture them in vitro or to find another method of induction of the transgene expression.

### Enhanced resistance against herbivores with anthocyanin content increased

Anthocyanins is one class of flavonoid compounds, and flavonoids are polyphenolic phytochemicals that constitute a large group of secondary metabolites in plants. Anthocyanin biosynthesis related to flavonoid biosynthesis produces important compounds for the defense against insects and pathogens (Dooner et al. 1991; Moctezuma et al. 2014). Although there have been relatively few reports concerning resistance against herbivores in *Lc*-transgenic plants, red leaves have been suggested by Hamilton and Brown (2001), to function as a visual warning to herbivores. Owing to increased amounts of defensive compounds in red leaves, the leaves containing higher concentrations of anthocyanins incur less natural herbivory. Additionally, the red leaves provide an effective chemical defense against larvae (Cooney et al. 2012) and anthocyanins in red leaf, as secondary metabolites, can function in plant defense against herbivores (Onkokesung et al. 2014). Analogous report showed that red kidney bean *Sayyad* was the most unsuitable host for feeding cotton bollworm (*H. armigera.common*) among common bean *Talash* and white kidney bean *Pak* (Namin et al. 2014). Similarly, transgenic plants that produced more anthocyanin exhibited a higher level of resistance against environmental stresses owing to their altered physiology (Flachowsky et al. 2010). Our study showed that the foreign *Lc* gene was not only expressed in cotton but also increased the anthocyanin content in transgenic leaves, and led to the green leaf changing to red or purple. In feeding experiment, cotton bollworms disfavor consuming red transgenic leaves, but ate green leaves. This is likely due to the higher anthocyanin in fresh leaves, which has been reported in the past to minimize herbivory (Cooney et al. 2012). The statistical analysis of insect weights showed that there was a significant difference between insects who fed on transgenic leaves and those fed on non-transgenic leaves (*P* < 0.01). This is most attributable to the accumulation of anthocyanin in the *Lc*-transgenic leaves. One aspect, cyanidin-3-β-glucoside is an important resistance factor in leaves that acts against the budworm and provides a potential basis for achieving insect resistance (Hedin et al. 1983). Cyanidin chloride and cyanidin-3-glucoside chloride slowed the growth of larvae when added to insect diet (Johnson et al. 2010). In another aspect, condensed tannin (polymeric anthocyanin) is also an important factor in plant–herbivore interactions (Furstenburg and van Hoven 1994; Boeckler et al. 2011). In plants, tannins serve to increase insect resistance by inhibiting several digestive enzymes, including proteases, pectinases, amylases, cellulases and lipases, as well as reducing protein digestibility. And these enzyme-inhibiting components decrease the conversion rate of food in larvae when reared on red kidney bean (Namin et al. 2014). Therefore, the resistance ability against herbivores, which was enhanced in *Lc*-transgenic leaves, was mainly attributable to the increased total anthocyanins.

In conclusion, based on our results, *Lc* alone is sufficient to trigger anthocyanin synthesis in different cell types in cotton under light conditions, and also increased resistance against bollworm. This may provide an alternative control method for cotton bollworms. Additionally, breeding a red fiber cotton variety through the regulation of anthocyanin biosynthesis in cotton fiber may be plausible in the near future.
